# Transcriptional precision and accuracy in development: from measurements to models and mechanisms

**DOI:** 10.1242/dev.146563

**Published:** 2017-11-01

**Authors:** Lital Bentovim, Timothy T. Harden, Angela H. DePace

**Affiliations:** Department of Systems Biology, Harvard Medical School, Boston, MA 02115, USA

**Keywords:** Precision, Accuracy, *Drosophila* development, Embryo, Transcriptional regulation, Modeling

## Abstract

During development, genes are transcribed at specific times, locations and levels. In recent years, the emergence of quantitative tools has significantly advanced our ability to measure transcription with high spatiotemporal resolution *in vivo*. Here, we highlight recent studies that have used these tools to characterize transcription during development, and discuss the mechanisms that contribute to the precision and accuracy of the timing, location and level of transcription. We attempt to disentangle the discrepancies in how physicists and biologists use the term ‘precision' to facilitate interactions using a common language. We also highlight selected examples in which the coupling of mathematical modeling with experimental approaches has provided important mechanistic insights, and call for a more expansive use of mathematical modeling to exploit the wealth of quantitative data and advance our understanding of animal transcription.

## Abstract (precis)

**Summary:** This Review highlights how high-resolution quantitative tools and theoretical models have formed our current view of the mechanisms determining precision and accuracy in the timing, location and level of transcription in the *Drosophila* embryo.

## Introduction

In animals, changes in gene regulation are associated with phenotypic changes within and between species (Chen and Rajewsky, 2007; Gompel et al., 2005; McGregor et al., 2007; Simpson, 2007; Wittkopp et al., 2008). To decipher how changes in gene regulation impact organismal phenotypes we must answer a fundamental question: which features of gene regulation confer changes in fitness? This question can be considered qualitatively and quantitatively. Qualitatively, it is important to turn the right genes on in the right place and at the right time, and to keep them off otherwise (St Johnston and Nüsslein-Volhard, 1992). Quantitatively, this question is much more complicated. First, multiple quantitative features of gene regulation may be important, such as the timing, location and level of transcript production. Second, for each feature we must define the ranges within which variation is tolerated and the ranges for which variation has phenotypic consequences (Harton and Batchelor, 2017). Finally, it may also be crucial to coordinate these features across multiple genes (Lagha et al., 2012). How quantitative features of transcription impact phenotype dictates how sequence variation can accumulate in regulatory DNA and thus determines the pool of regulatory sequence variants that are available for natural selection.

As a developmental biologist, a biological physicist and a systems biologist, our respective backgrounds and sometimes conflicting perspectives motivated this Review. Quantitative *in vivo* measurements of animal transcription are a recent innovation and are now being widely deployed (Ferraro et al., 2016b). However, we have not yet developed an adequate conceptual or computational framework to link relevant molecular mechanisms, which are still largely defined qualitatively (Lagha et al., 2012), to quantitative features of transcription, such as precision, accuracy, robustness, plasticity and stability. The definitions of such quantitative features are also not always clear or uniformly applied in the literature and across disciplines. Take for example the term ʻprecision': in the physical sciences, precision narrowly refers to statistical variation (noise) in a system, such as the standard deviation of a normally distributed variable; in biological studies of transcription, however, the term is used more broadly and can refer to statistical variation as well as what physicists call ʻaccuracy' (the difference between the population average and a target value) (Elowitz et al., 2002; Lagha et al., 2012). When we began writing, we did not realize that we were all using the word ʻprecision' in different ways, and this significantly hindered our ability to think together about how to link measurements to mechanisms. We realized that, colloquially, precision and accuracy are often synonyms that are used interchangeably and that this varies across languages and scientific disciplines; this confusion makes clear scientific definitions even more important.

Here, we present precision and accuracy as core definitions upon which other features, such as robustness, plasticity and stability, depend (see Box 1 for definitions). We highlight that, depending on your goal, you can consider the precision or accuracy of a given transcriptional feature, such as the timing, level or position of transcription, to derive mechanistic insights. We review recent experimental studies of the *Drosophila melanogaster* embryo that use quantitative techniques to interrogate the mechanisms of transcription that control timing, level and location. We also suggest that identifying the molecular mechanisms underlying transcriptional precision and accuracy will require the construction of mathematical models rooted in physics. Such models can produce falsifiable predictions based on widely held assumptions about proposed mechanisms (Garcia et al., 2010). Ultimately, models will be the lens through which we can examine the mechanistic underpinnings of quantitative transcriptional features and thus determine the raison d’être of gene regulatory pathways.

Box 1. Definitions of features of transcriptional regulation **Transcriptional precision.** High transcriptional precision refers to low relative variation in the timing, level or spatial location of the transcription of a gene between individual cells or embryos of the same population.

**Transcriptional accuracy.** The expression of a gene at a specific time, to a specific level or in a specific location. When quantitative features (such as levels, timing and location) of the transcriptional output differ from their average target value, the transcriptional output is less accurate. The target value can refer, for example, to the average value measured in the wild type. In the field of transcription, biologists often use the term ‘accuracy’ synonymously with the term ‘precision’.

**Robustness.** Robust transcription is the faithful execution of precise and accurate transcription when confronted with a perturbation, usually environmental or genetic.

**Stability.** Stability refers to the tolerance of a feature to a noisy and stochastic molecular environment (e.g. producing a precise transcriptional output in a noisy environment).

**Plasticity.** We refer to transcriptional plasticity as the ability of a regulatory mechanism to change, while transcriptional accuracy and precision are conserved. For example, when the type or number of transcription factor binding sites within an enhancer changes during evolution, while the output driven by it does not.

## Defining precision and accuracy in transcription

Transcription can be characterized by the following features: (1) the location of transcription, i.e. the expression of a gene in a specific spatial location within an organism; (2) the level of transcription, i.e. the number of transcripts within a given cell at a particular time; and (3) the timing of transcription, i.e. when the expression of a particular gene is turned on/off; this can be defined in absolute time, or relative to a particular developmental event (such as the onset of cellularization), or relative to other cells within an organism. When considering these features, we can also define the precision and the accuracy of transcription: precision in transcription refers to minimizing variation in these features between individual cells or embryos within the same population; accuracy describes how close the average values of these features are between different populations, such as wild-type and perturbed embryos.

It is useful to distinguish between precision and accuracy because they can lead to distinct mechanistic insights. For example, we often use measurements of transcriptional accuracy to confirm that a putative target is regulated by a specific transcription factor (TF). This usually involves perturbing the TF – manipulating its level or binding site sequence – and measuring changes in the transcriptional output (Staudt et al., 2006). This general approach has been applied to many regulatory proteins and was used to infer gene regulatory networks in development, often by also using computational models (Barsi et al., 2015; Kozlov et al., 2012). Alternatively, measuring transcriptional precision (i.e. cell-to-cell variability in nascent transcript production) can reveal the number of regulated steps in a process (Choubey et al., 2015). In this case, to gain mechanistic insight from measuring precision before and after perturbation, a mechanistic model of the underlying process is required.

Although defining precision and accuracy is relatively straightforward, linking changes in these parameters to organismal phenotype is not. Development requires the accurate specification of cell fate during differentiation, even when it is subject to genetic and environmental perturbations (Perrimon et al., 2012). Because transcription is central to differentiation, precision and accuracy in transcription are inherently linked to the reproducibility and accuracy of development. However, we do not yet know the nature nor the strength of the connection between the quantitative features of transcription and the accuracy of development. There are two main reasons for this. First, multiple mechanisms aside from transcription contribute to the accuracy and reproducibility of development. These include mechanisms of post-transcriptional regulation (Weil, 2015), and important compensatory mechanisms at the level of cellular networks, cell-to-cell interactions and tissue mechanics, among many others (Chalancon et al., 2012; Little et al., 2013; Raj et al., 2010). Second, multiple quantitative features of transcriptional regulation may be relevant (see Box 1). Each of these features may have distinct error tolerances and thus different phenotypic consequences for development.

A good example of how the accumulation of subtle changes in transcriptional output can lead to significant changes at the organismal level comes from the study of the *Shavenbaby* (*Svb*; *ovo* – FlyBase) gene in *Drosophila* (Frankel et al., 2011; McGregor et al., 2007). *Svb* encodes a TF that regulates the morphogenesis of microtrichiae – the small hairs found on the larval surface. It has been shown that the accumulation of multiple mutations in the regulatory DNA of this gene, each with a quantitative effect, substantially alters the timing and levels of *Svb* expression; together, this results in changes in the patterning of microtrichiae and hence morphological differences between multiple *Drosophila* species (Frankel et al., 2011; McGregor et al., 2007). Similarly, phenotypic changes in abdominal pigmentation within an African *D. melanogaster* population resulted from a combination of mutations in the regulatory DNA of the *ebony* gene, each of which exerts a small effect (Rebeiz et al., 2009). These studies point to the potential organismal consequences of even subtle quantitative changes in transcriptional output.

## Mathematical models are useful for understanding transcriptional precision and accuracy

The results described above emphasize that to link changes in regulatory DNA sequence to changes in organismal phenotype we must elucidate the mechanisms that control the precision and accuracy of transcription. Theoretical frameworks that reflect what is observed experimentally can help elucidate such mechanisms (Coulon et al., 2013; Garcia et al., 2010). This strategy was pioneered in bacteria, where mathematical models of gene regulation have been used to successfully predict experimental measurements of transcription in space and time and under various perturbations (Bintu et al., 2005; Brewster et al., 2012, 2014; Choi et al., 2008; Elowitz et al., 2002; Garcia and Phillips, 2011; Kuhlman et al., 2007; So et al., 2011; Taniguchi et al., 2010), and has been reviewed previously (Ay and Arnosti, 2011; Sanchez et al., 2013). Importantly, when experiments contradict the predictions of mathematical models, this points to more biology for us to discover; discrepancies inspire new concepts, model revision and further experiments. The showpiece for this approach has been the long-studied *lac* operon (reviewed by Garcia et al., 2010). Some argue that it is impossible to faithfully represent highly complex molecular processes such as eukaryotic transcription by ʻsimple' mathematical models like that used for the *lac* operon. Indeed, many experimentally trained biologists believe a model should take into account all the relevant molecular components and capture all known mechanistic features of a system. However, this quickly leads to highly complex models that are difficult to validate. Capturing all known information is not the purpose of a simple model. Instead, simple models are built to articulate our assumptions and translate our hypotheses into a mathematical framework. We can then directly test the assumptions on which the model is built, identify the important variables and make falsifiable predictions (Gunawardena, 2014a; Möbius and Laan, 2015; Phillips, 2015).

To retain simplicity when modeling complex processes, models can contain variables that encapsulate multiple molecular aspects of a biological system (e.g. Estrada et al., 2016). These types of aggregate variables still point to relevant molecular features and they can be unpacked by refinement of the model and further experiments. Therefore, models are useful even when not all the molecular players are included (perhaps because they are unknown) and especially when mechanisms cannot be understood intuitively. The process of building models can also clarify our thinking and gives a starting point for discussing our work with others. We might disagree on what a cartoon means and how it will behave under perturbation, but a mathematical model is a logical machine that yields a defined outcome. We can thus focus our discussion on the assumptions of the model and interesting discrepancies between the model predictions and experimental data. Mathematical models are thus a way for us to calibrate our degree of surprise about an experimental result and the robustness of our current concepts.

**Fig. 1.**
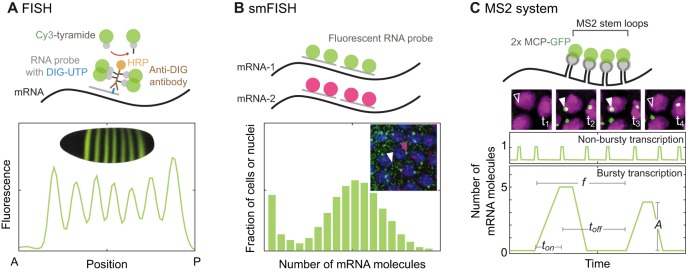
**Quantitative methods to measure transcription in living organisms.** (A) Fluorescence *in situ* hybridization (FISH) labels target mRNA molecules in fixed cells through tyramide signal amplification. Digoxigenin-labeled (DIG-UTP) RNA probes are hybridized to target mRNA molecules and bind anti-DIG antibodies conjugated to horseradish peroxidase (HRP). This enzyme generates tyramide radicals that can then covalently react with nearby residues. The inset shows the *eve* expression pattern in *Drosophila* embryos, as generated by combining many FISH experiments into an expression atlas (Fowlkes et al., 2008); the plot depicts a line trace along the anteroposterior (AP) axis of the embryo (*n*=30). (B) Single-molecule FISH (smFISH) enables the counting of individual mRNA molecules in fixed cells or nuclei. Fluorescently labeled DNA oligonucleotides (red and green circles) are hybridized to specific mRNA molecules, rendering bright fluorescent spots both at active sites of transcription (inset, magenta arrowhead) and elsewhere in the cell (inset, white arrowhead). DAPI-stained DNA is in blue. To analyze these data, a probability distribution of the number of molecules per cell/nuclei can then be fitted with a model to extract parameters (as depicted in Fig. 2). Image was generously provided by Shawn C. Little (University of Pennsylvania). (C) The MS2 system allows real-time dynamic measurements in live cells and organisms. Genetically encoded fluorescently tagged coat proteins (MCP-GFP, green circles) bind to repeats (8 to 128) of a stem-loop sequence inserted in the gene sequence of interest. Other similar coat protein systems are also used, although MS2 is the most common (Lange et al., 2008; Larson et al., 2011). Shown here is the same field of view over time, depicting nuclei (magenta) with fluorescent MS2 signal (green) at active sites of transcription (solid arrowhead); sites before and after transcription takes place are indicated (open arrowhead). These data allow a coarse-grained characterization of transcription kinetics; the frequency (*f*), amplitude (*A*), duration (*t_on_*), and time between transcription (*t_off_*) can be measured to determine whether a gene is expressed constitutively (i.e. non-bursty, above) or in bursts (below).

The types of quantitative measurements that were required to implement this approach when studying bacterial transcription are more difficult in higher organisms. For example, the single-molecule techniques that yield exquisite detail for bacterial transcription (e.g. Friedman and Gelles, 2012; Harden et al., 2016; Revyakin et al., 2004; Zhang et al., 2014) remain challenging for eukaryotic transcription (Chen and Larson, 2016). However, powerful imaging techniques that provide dynamic, quantitative data in both cells and intact organisms are available and well suited to study active transcription in animals (Fig. 1) (Gregor et al., 2014). For example, single-molecule fluorescence *in situ* hybridization (smFISH), fluorescence correlation spectroscopy, single-particle tracking, and genetically encoded fluorescent RNA labeling, including the popular MS2/MS2 coat protein system (reviewed by Abbaszadeh and Gavis, 2016; Yao, 2017), are gaining wide use in the field (Abbaszadeh and Gavis, 2016; Bothma et al., 2015; Ferraro et al., 2016a; Fukaya et al., 2016; Garcia et al., 2013; Larson et al., 2013; Lenstra et al., 2015; Lucas et al., 2013; Tantale et al., 2016). However, if they are to provide an understanding of the molecular mechanisms that ensure precise and accurate transcription it is imperative they are informed by mathematical models (Coulon et al., 2013; Gunawardena, 2014b).

**Fig. 2.**
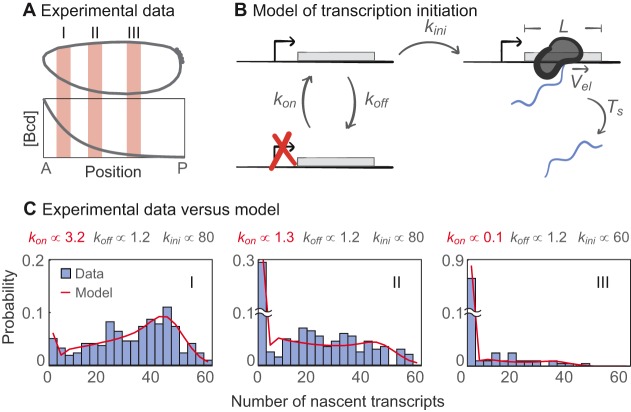
**A simple model to elucidate transcription kinetics from experimental data.** (A) Two hours after fertilization, *hb* expression was measured within individual nuclei located in three regions of the embryo (I, II and III; orange) by smFISH to nascent *hb* transcripts; each region contains different concentrations of Bcd protein along the AP axis. (B) Schematic of a stochastic model of transcription initiation kinetics used to fit the data in C, including the length of the gene (*L*), Pol II elongation speed (*V_el_*), and the time for RNA release (*T_s_*). (C) Experimental distribution of nascent RNA (blue bars) within regions I, II and III, with a theoretical fit of the model shown in red*.* To capture the changes in nascent RNA distribution across the embryo, it was sufficient to change a single parameter in the model, *k_on_*, which physically modulates the frequency of transcriptional bursts, while the parameters for the duration (*k_off_*) and amplitude (*k_ini_*) of bursts remain almost unchanged. Figure adapted with permission from Xu et al. (2016).

A study by Xu et al. on transcriptional regulation of the *hunchback* (*hb*) gene in *Drosophila* embryos provides an excellent example of how mathematical models can be used to interpret quantitative data. Using smFISH (Xu et al., 2015), the authors dissected the kinetics of *hb* transcription and made mechanistic inferences about the function of the TF Bicoid (Bcd) (Xu et al., 2016). They showed that the impact of Bcd on expression kinetics can be explained by modulating the rate at which the promoter switches from a silent ʻoff' state to a transcriptionally competent ʻon' state, using a simple theoretical model of the stochastic kinetics of expression (Fig. 2). The description of Bcd can now go beyond that of an ʻactivator' to include details of its activating function, much the same way as bacterial TFs have been characterized in detail (e.g. Artsimovitch and Landick, 2002; Ha et al., 2010; Harden et al., 2016; Mooney et al., 2009). A clear next step is to extend this approach to encompass other eukaryotic TFs. Activator-dependent transcription has been detailed by decades of biochemistry, and it is clear that activators can influence different regulated steps of transcription (Fuda et al., 2009). Identifying signatures of specific activities from imaging data and mathematical models may help characterize larger numbers of eukaryotic TFs. Eventually, the field might crack the elusive cis-regulatory code (Yáñez-Cuna et al., 2013) by deciphering how TFs work together to control gene expression (Keung et al., 2014; Scholes et al., 2016; Stampfel et al., 2015).

## Mechanisms proposed to control transcriptional precision and accuracy

As we highlight below, various mechanisms have been proposed to impact the location, levels and timing of transcription. We note that each one of these features can be characterized by their precision (or noise) and accuracy (deviation from the average), but that this is not how most results are discussed in the literature. Most of the studies we describe address transcriptional accuracy, for example by perturbing a component genetically and measuring the effect on an average value. However, with the recent increased use of quantitative tools, the number of studies that analyze both precision and accuracy to gain mechanistic insights is growing. We also note that some mechanisms are proposed to regulate multiple features. For example, functional interactions between shadow enhancers have been shown to influence the location, level and timing of transcription. However, it is not yet clear how control of the location, level and timing of transcription is mechanistically intertwined or to what extent, if any, these features can be modulated independently. Finally, it should be noted that we focus our discussion on gene regulation via transcriptional enhancers.

Enhancers are 100-1000 bp sequences that contain clusters of TF binding sites and typically activate transcription from a core promoter by recruiting TFs to the site of transcription (Long et al., 2016; Spitz and Furlong, 2012). They are usually located at a distance from the gene itself, as opposed to promoters which are located next to the gene and allow transcription initiation (Kim and Shiekhattar, 2015). Importantly, enhancers control spatial and temporal gene activation during development and are the primary regulator of differential gene expression (Ong and Corces, 2011). Here, we discuss gene regulation by enhancers, enhancer-promoter interactions, and locus-level integration of information from multiple enhancers. We do not address genome-scale mechanisms, such as chromosome conformation or nuclear localization; these have been recently reviewed elsewhere (de Laat and Duboule, 2013; Long et al., 2016; Sexton and Cavalli, 2015; Shachar and Misteli, 2017). We also focus on examples from *Drosophila*, as this is our area of expertise, although similar quantitative studies have also been conducted in other organisms and some have been recently reviewed in the context of bacterial (Browning and Busby, 2016) and mammalian (Zhao et al., 2016) transcription. Furthermore, it should be noted that only some of the experiments we describe are successfully coupled to computational models; this emphasizes the wealth of opportunities to translate proposed mechanisms of animal transcription into mathematical models, which can then be experimentally tested.

### Mechanisms that regulate the location of transcription

#### Combinatorial control by cis-acting elements directs expression patterns

Within a single enhancer, regulatory proteins, such as TFs and co-factors, collaborate to control the spatial specificity of expression. The field has long searched for the rules that govern how the number, affinity and arrangement of TF binding sites affect transcript production; such rules are referred to as ʻcis-regulatory grammar'. Several models for cis-regulatory grammar have been proposed. While these models have primarily focused on the accuracy of pattern position, with dynamic data they can now begin to address transcriptional noise/precision. These conceptual models range from the rigid requirement that TF spacing and orientation must be preserved (Thanos and Maniatis, 1995) to the idea that it is sufficient to simply recruit a certain set of TFs (Kulkarni and Arnosti, 2003; Long et al., 2016).

Multiple mathematical models have tested the rules of cis-regulatory grammar by relating regulatory DNA sequence to expression patterns in multiple systems, including *Drosophila* embryos. These models build on the thermodynamic framework used for prokaryotic transcription by including terms to account for additional complexities in metazoan transcription, such as variations in TF binding site affinities (He et al., 2010; Janssens et al., 2006; Segal et al., 2008) and inhibition by repressor proteins (He et al., 2010; Janssens et al., 2006; Zinzen et al., 2006). Collectively, these models suggest that TF occupancy on enhancers can be a rate-limiting step in initiation (Zinzen et al., 2006) and that repressors can act locally to ʻquench' the function of activators (He et al., 2010; Janssens et al., 2006). However, newer high-resolution dynamic measurements make it clear that modeling TF binding and RNA polymerase II (Pol II) recruitment to regulatory DNA at equilibrium (which forms the basis of the prokaryotic models cited above) is not sufficient to capture the important dynamics of gene regulation (Garcia et al., 2010; Sanchez et al., 2013), and new classes of stochastic models that can capture noise are being developed (Coulon et al., 2010; Sánchez and Kondev, 2008).

The spatial position of expression patterns is also influenced by interactions between enhancers that exhibit overlapping spatiotemporal activity, usually referred to as shadow enhancers (Barolo, 2012; Hong et al., 2008). The number of genes thought to be regulated by shadow enhancers is growing quickly, both in *Drosophila* (Cannavò et al., 2016) and in human cells (Adam et al., 2015; Hnisz et al., 2015). Interrogating a mathematical model of *even skipped* (*eve*) enhancer function uncovered shadow enhancers in the *eve* locus (Staller et al., 2015), and models have predicted shadow enhancers in other genes as well, although they have not been experimentally validated (Kazemian et al., 2010). Shadow enhancers can ensure robust transcription under perturbations by environmental conditions or genetic background (Dunipace et al., 2011; Frankel et al., 2010; Perry et al., 2010). Shadow enhancers can also interfere with or repress each other's activity (Dunipace et al., 2011; El-Sherif and Levine, 2016; Garcia et al., 2013; Hang and Gergen, 2017; Lucas et al., 2013; Perry et al., 2011, 2012; Prazak et al., 2010), but general principles to predict their interaction have not yet emerged. Simple mathematical models that focus on how shadow enhancers compete with the promoter have been developed, and these explain a portion of existing experimental data (Bothma et al., 2015; Perry et al., 2011). However, promoter competition is not the only possible mechanism for shadow enhancer interaction, and others are actively being explored (Kim et al., 2009; Long et al., 2016; Samee and Sinha, 2014; Scholes et al., 2016). For example, enhancers that are found in close proximity and share the same pool of TFs may modulate local TF concentrations, which can influence TF binding kinetics (Crocker et al., 2017b preprint). Another possibility is that shadow enhancers regulate different kinetic steps of the transcription cycle (Scholes et al., 2016), as further discussed below in the section on shadow enhancers.

#### The formation of sharp boundaries

**Fig. 3.**
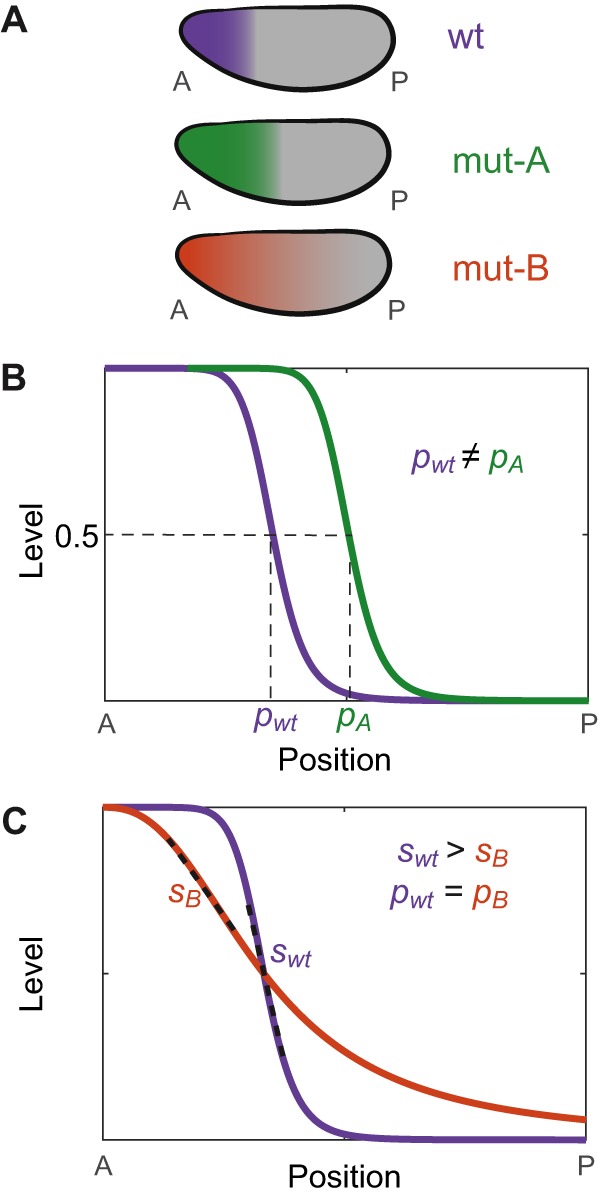
**Precision and accuracy in the spatial location of transcription.** (A) Examples of differences in the expression pattern of a gene between wild-type (wt) and perturbed/mutant (mut) early *Drosophila* embryos. (B) Accuracy in the spatial location of transcription can be assessed; for example, by comparing the expression pattern border position (*p_i_*), here determined by the half-maximum expression level along the AP axis. In mut-A, the border position is different from that of the wt and therefore lacks accuracy. (C) Precision in location can refer to reduced variation in expression levels near the expression pattern border, increasing the sharpness of the border. The maximum slope of the expression level versus position curve (i.e. the steepness, dashed lines) is a quantitative measurement of the sharpness of the expression pattern border. In mut-B, the steepness of the boundary (*s_i_*) is less than that measured in the wt embryo. This leads to greater cell-to-cell variability in expression levels near the pattern border and a less precise or broader border region. In mut-B, as opposed to mut-A, the position of the expression pattern border, as defined above, is accurate.

Beyond getting a gene expressed in the correct region of an embryo, it can also be crucial to obtain sharp boundaries on that region. The step-like expression pattern of *hb* is a flagship model for studying the formation of sharp expression boundaries (Fig. 3). Anterior *hb* expression is regulated by the exponentially distributed activator Bcd (Driever and Nüsslein-Volhard, 1988a,b; Struhl et al., 1989) and is one of the best-studied patterns in the fly embryo (Gregor et al., 2007a; Margolis et al., 1995; Struhl et al., 1989). The pattern is known to be directed by the accumulation of *hb* mRNA transcripts in the early embryo rather than by post-transcriptional processes, and is widely thought to be due to cooperative binding of Bcd to the *hb* P2 enhancer. However, recent studies indicate that the molecular details underpinning sharpness are yet to be worked out (Estrada et al., 2016; Garcia et al., 2013; Lucas et al., 2013).

The first studies to use MS2 reporter genes to image transcription in live embryos examined the *hb* P2 promoter and proximal enhancer (Garcia et al., 2013; Lucas et al., 2013). Garcia et al. showed that during pattern formation, once activated, cells continually express *hb* for the duration of the pattern. They also found that including the Pol II loading rate and the activation time window in a quantitative model of expression is not sufficient to explain the observed sharp boundary. Instead, an additional physical parameter – the stochastic activation of cells near the boundary region – is necessary to explain the sharpness of the boundary in their model. A study by Desponds et al. (2016) described how to infer simple kinetic models of transcript initiation from MS2 data in early *Drosophila* embryos, and this should serve as a guide for extracting more detailed kinetic parameters from these types of data.

As mentioned above, cooperative pairwise binding of Bcd to the *hb* proximal enhancer has long been considered to be the primary mechanism for generating a sharply delineated *hb* expression pattern (Gregor et al., 2007b). However, mathematical modeling suggests that pairwise cooperative Bcd binding is not sufficient to explain the sharp on/off boundary, implicating other mechanisms such as interactions with co-factors or chromatin in generating such boundaries (Estrada et al., 2016; Lopes et al., 2008). The model presented by Estrada et al. (2016) can capture the Bcd-directed formation of a sharp *hb* boundary by including either information integration or energy expenditure. Information integration refers to a ʻhigher order cooperativity' term wherein multiple non-adjacent Bcd molecules can influence one another; this cooperativity may arise from interactions with co-regulators such as the Mediator complex or CBP/p300. Alternatively, energy expenditure that keeps the system away from equilibrium can achieve sharp expression boundaries; energy is burned by a number of well-established molecular pathways involved in transcription, including post-translational modifications of histones or the transcriptional machinery itself (Clapier et al., 2017; van der Knaap and Verrijzer, 2016; Varga-Weisz et al., 1997; Wright et al., 2016). These concepts are not considered in the classic mathematical models of transcription developed for bacteria.

Repressors may also be involved in regulating the formation of sharp boundaries, as shown for *hb* (Chen et al., 2012; Manu et al., 2009a,b) and in synthetic systems (Crocker et al., 2017a). Crocker et al. (2017a) tested the difference between overlapping and tandem arrangements of activator and repressor binding sites, and demonstrated that overlapping binding sites, which are common in developmental enhancers, produce sharper boundaries. The ability to isolate TF function from a native sequence, which contains binding sites for many factors that may exhibit context-dependent function, allows for highly controlled study of TF function (e.g. Fakhouri et al., 2010). Synthetic approaches also provide a test of our understanding; however, predicting and building a regulatory sequence from scratch has not yet been successful, indicating that we have more to learn (Johnson et al., 2008; Vincent et al., 2016). Quantitative measurements and models are likely to be helpful in this goal; ground-up synthetic approaches in prokaryotes have had a successful history when informed by mathematical modeling (Bintu et al., 2005; Choi et al., 2008; Choubey et al., 2015; Vilar et al., 2003; Yildirim and Mackey, 2003).

### Mechanisms that regulate the level of transcription

#### Modulating transcriptional bursts

**Fig. 4.**
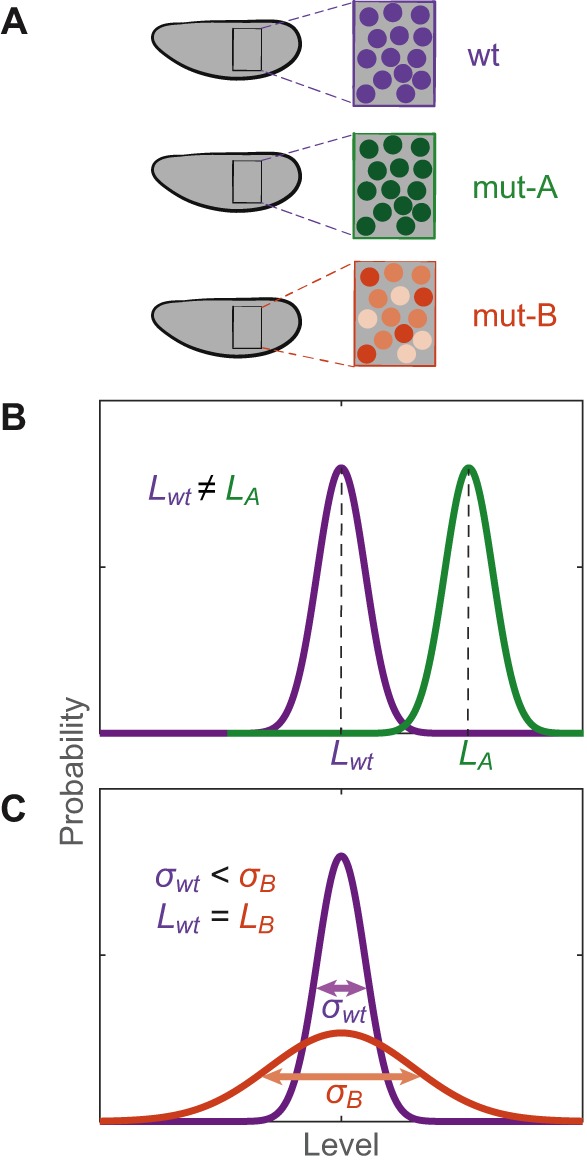
**Precision and accuracy in the level of transcription.** (A) Examples of differences in the level of transcription (i.e. the number of newly produced transcripts) of a gene between wild-type (wt) and perturbed/mutant (mut) early *Drosophila* embryos. (B) Accuracy in the level of transcription can be assessed; for example, by comparing the average level of nascent transcripts across a population of cells or nuclei (*L*). In mut-A, this value is different from the wt value and is not accurate. (C) Precision in transcript level can refer to the variation of transcript level within a population of cells/nuclei. Nuclei in the mut-B embryo exhibit more variation (*σ_i_*) than nuclei in the wt embryo and the transcript level is thus less precise. Since wt and mut-B embryos have the same *L* value, the mut-B expression level is accurate.

Transcription often occurs in bursts, whereby multiple mRNA molecules are synthesized consecutively, followed by a period of promoter inactivity (Lenstra et al., 2016; Lionnet and Singer, 2012; Sanchez and Golding, 2013). The rate of transcript production in a cell depends on burst amplitude (i.e. the number of transcripts being produced during the burst), duration (i.e. the time window when the promoter is active) and frequency (i.e. the time between two consecutive bursts) (Fig. 1C). Modulating each of these burst parameters can affect the accuracy and precision of transcript levels within cells (Fig. 4). In *Drosophila* embryos, smFISH of the Hox gene *Sex combs reduced* (*Scr*) indicates bursty expression (Paré et al., 2009); expression of an MS2 reporter driven by the *eve* stripe 2 enhancer is also bursty (Bothma et al., 2014). But what mechanisms drive these bursts of transcription?

It has recently been demonstrated that developmental enhancers can modulate burst frequency in *Drosophila* embryos (Fukaya et al., 2016). In this study, insulators, which change enhancer-promoter interactions by restricting DNA topology, were shown to reduce transcript level and increase precision by attenuating burst frequency. Enhancer modulation of burst frequency through promoter looping has also been shown in mammalian cells, where forced looping of the β-globin enhancer results in increased burst frequency, but not amplitude, of the β-globin gene. Interestingly, during erythroid maturation, both burst frequency and amplitude of the β-globin gene increase, indicating that in this case additional mechanisms other than looping regulate transcript levels (Bartman et al., 2016).

#### The physical interaction of regulatory elements

As indicated by the forced looping experiment discussed above, expression levels can be regulated by changing the extent of physical association between enhancers and promoters (Cai and Levine, 1995; Chopra, 2011; Fukaya et al., 2016). It is common practice to observe such interactions using chromosome conformation capture techniques (Cattoni et al., 2015; de Wit and de Laat, 2012; Ghavi-Helm et al., 2014). However, these techniques produce static pictures for the entire genome, averaged across many cells over time, although single-cell versions of these techniques are now emerging (Nagano et al., 2013; Ramani et al., 2017). To develop a clearer picture of the physical interactions between enhancers and promoters, live imaging techniques will be required and indeed are emerging. For example, Fukaya and colleagues challenged our current view of enhancer-promoter looping by showing that a single enhancer can activate two different promoters simultaneously (Fukaya et al., 2016). In addition, Chen et al. recently employed multi-color fluorescence microscopy with three fluorescently tagged proteins to visualize interactions between endogenous *eve* enhancers and a second *eve* promoter inserted 142 kb upstream of the *eve* locus (Chen et al., 2017 preprint). They concluded that stable enhancer-promoter interaction is a requirement for activation and continued expression, and that this interaction cannot be mediated by enhancer-bound TFs alone.

These advances in measuring and characterizing enhancer-promoter interactions make this a ripe area for the development of computational models. Indeed, modeling chromosome dynamics has had success in predicting complex cellular processes, such as mating-type switching in yeast (Avşaroğlu et al., 2016). This approach applied biopolymer models – the theoretical treatment of principal structures in living systems as semiflexible polymers (Broedersz and MacKintosh, 2014). A similar polymer model was applied explicitly to enhancer-promoter looping to provide mechanistic insight into recent experimental results on the role of looping in gene regulation (Doyle et al., 2014). However, polymer models alone cannot quantitatively predict the regulatory roles of looping without explicitly modeling the relationship between enhancer-promoter contact frequency and gene expression; this will require further work to elucidate the link between TF function and the quantitative features of transcription.

#### Shadow enhancers

**Fig. 5.**
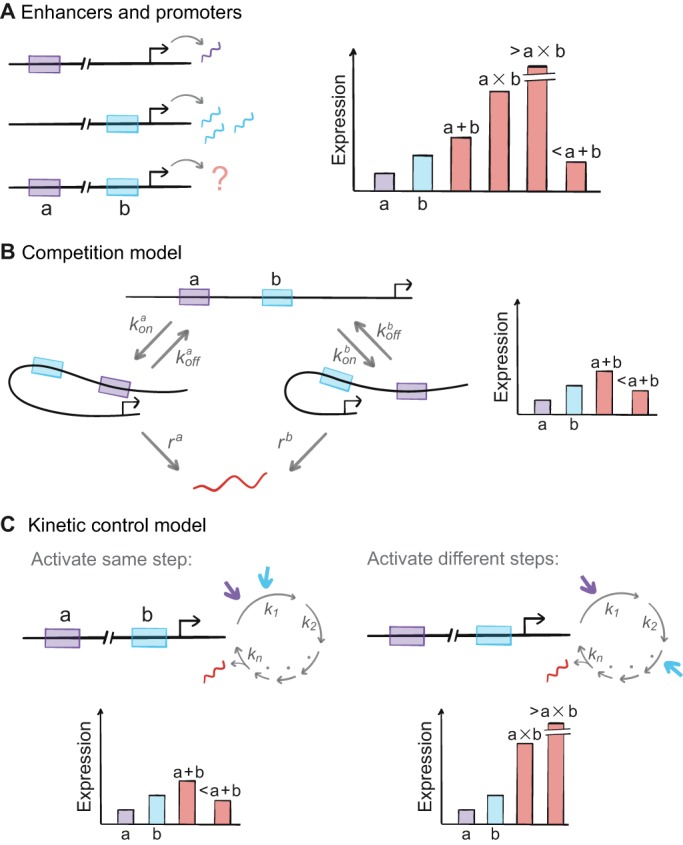
**How do multiple enhancers acting on the same gene combine to regulate expression?** (A) Different enhancers (a and b) can drive different levels of transcription from the same gene. The gene promoter is marked by a black arrow. Specifically for expression level, there is no single model for predicting how the activity of enhancers will combine to regulate transcription (orange), and various outcomes are theoretically possible (as depicted in the bar chart), although different models have been proposed as detailed in B and C. (B) The ‘competition model' from Bothma et al. (2015) is based on the hypothesis that combinatorial control depends on enhancer strength, which reflects the frequency and duration of promoter-enhancer interactions. This model is based on the assumptions that the promoter can interact with a single enhancer at a given time and these interactions are at equilibrium. *k_on_* and *k_off_* are the rates of promoter engagement and disengagement, respectively, and *r^i^* is the rate of mRNA production when the promoter is engaged with a specific enhancer. The ‘competition model’ predicts that enhancers that activate their cognate promoter infrequently (i.e. with a sufficiently small *k_on_* and a sufficiently large *k_off_*) combine additively, whereas enhancers with frequent activation (i.e. with a sufficiently large *k_on_* and a sufficiently small *k_off_*) combine subadditively, as depicted in the bar chart. (C) An alternative model, termed the ‘kinetic control model’, can explain the diverse observations that have been reported for the combined activity of multiple enhancers (Scholes et al., 2016). This model, initially applied to transcriptional regulation by individual TFs, may extend to enhancer-level regulation. It proposes that enhancers (via the TFs that bind them) can regulate multiple rate-limiting steps in the transcription cycle, such as transcription initiation and transition to elongation (individual steps in the cycle are depicted by gray arrows and *k_n_* marks their respective rate constants). They can either activate or repress the same or different steps in the cycle. Importantly, this model eschews one key assumption of traditional equilibrium models developed for prokaryotes: that transcription has a single rate-limiting step and this step is the target of all regulation. Here there are examples for two enhancers that activate (purple and blue arrows) either the same step (left) or different steps (right). In contrast to the competition model, the kinetic control model can generate a wider range of predicted expression levels (possible outputs for each example are represented in the bar chart).

Pairs of shadow enhancers for different developmental genes can drive unpredictable levels of output: equal to, greater, or less than the sum of the output from individual enhancers in isolation (Fig. 5A) (Bothma et al., 2015). For instance, the combined output driven by *hb* shadow enhancers varies between subadditive in the presence of saturating levels of the activator Bcd, and additive when Bcd protein levels are low. Conversely, output driven by the *knirps* shadow enhancers, which activate transcription initiation at a lower rate than the *hb* enhancers, varies between greater-than-additive to additive for different times prior to gastrulation. Bothma et al. (2015) proposed a mathematical model based on competition between shadow enhancers for the promoter, assuming only one enhancer can interact with the promoter at a time. In this model, the combinatorial effect of enhancers depends on their interaction strength: ‘weak’ enhancers do not often interact with the promoter and therefore do not interfere with one another, allowing their effect to be additive. By contrast, ‘strong’ enhancers frequently interact with the promoter and therefore interfere with each other's activity, leading to non-additive or subadditive effects (Fig. 5B). Notably, this model cannot explain a superadditive output, as was observed for *knirps*. This discrepancy therefore calls for additional mechanisms. Alternatively, a more general model might be required, such as kinetic control, whereby shadow enhancers regulate different kinetic steps of the transcription cycle, as suggested by Scholes et al. (2016) (Fig. 5C). One intriguing possibility is that multiple enhancers can simultaneously interact with the same promoter. As mentioned above, the reciprocal case, where a single enhancer can interact simultaneously with two promoters, was recently demonstrated (Fukaya et al., 2016).

Measuring the combined output of orthologous pairs of shadow enhancers can reveal whether precision and accuracy are conserved. *Kruppel* (*Kr*), a key patterning gene in *Drosophila* embryos, is regulated by a pair of shadow enhancers (Hoch et al., 1990), and Wunderlich et al. (2015) demonstrated that the expression level of *Kr* is highly conserved across three different *Drosophila* species, while the level of expression driven by each individual enhancer differs. In this case, transcriptional accuracy is conserved, suggesting that maintaining specific levels of this gene is crucial, but that there are multiple ways to generate the same level using shadow enhancers that differ in their individual activity. Wunderlich et al. (2015) also showed that the *Kr* proximal and distal enhancers are activated by different sets of TFs. The *brinker* (*brk*) shadow enhancers are also regulated by different TFs, leading to an interesting hypothesis that shadow enhancers are not simple duplications but instead work on distinct steps of the transcription cycle or at distinct times (Dunipace et al., 2013).

### Mechanisms that regulate the timing of transcription

#### Promoters can induce synchrony

**Fig. 6.**
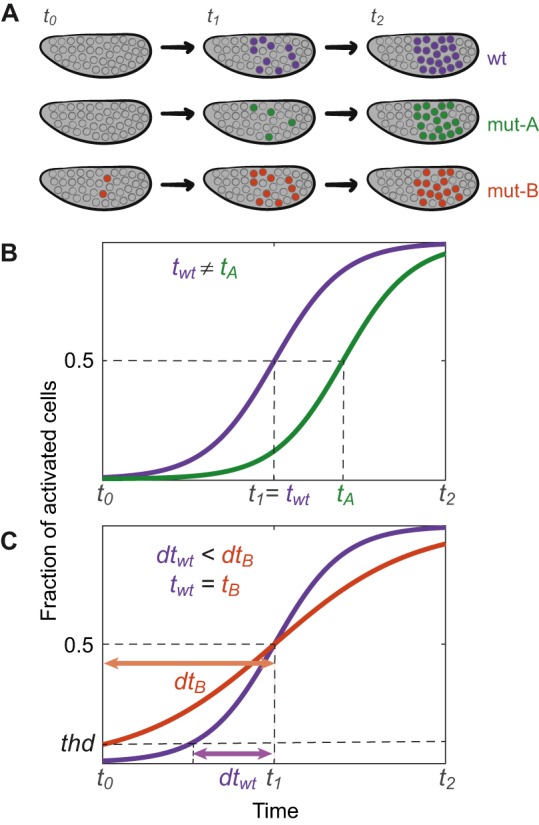
**Precision and accuracy in the timing of transcription.** (A) Examples of differences in the timing of transcriptional activation of a gene between wild-type (wt) and perturbed/mutant (mut) early *Drosophila* embryos. (B) Transcriptional synchrony in a population of cells can be determined by measuring the time when transcription is activated in a given fraction of cells, e.g. *t_wt_* and *t_A_* are the times when 50% of wt and mut-A cells are active, respectively. Accuracy in the timing of transcription can refer to changes in the average value of this time, as demonstrated between mut-A and wt embryos. (C) Precision in timing can be measured in absolute time, or relative to a developmental event, or relative to other cells within an organism. Synchrony of the population of cells can be determined by measuring the time between initial activation (when the signal surpasses a threshold, *thd*) and *t_50_* (*dt_i_*). Here, activation in mut-B is less synchronized than in the wt (*dt_B_* is larger than *dt_wt_*) and therefore the timing of activation is less precise. Since wt and mut-B embryos have the same *t_50_* value, there is no change in the accuracy of the timing of activation. It should be noted that temporal dynamics can affect transcript levels in cells/nuclei. For example, earlier transcription activation can result in the accumulation of more transcripts over time.

Pol II promoter-proximal pausing, wherein transcription stalls after synthesizing 30-50 nucleotides of nascent RNA, is a pervasive feature of gene regulation in higher eukaryotes, and is thought to be important for the regulation of stimuli-responsive and developmental genes (Adelman and Lis, 2012; Robinson et al., 2016). Because promoter-proximal paused Pol II is enriched at many important developmental genes in *Drosophila* (Boettiger and Levine, 2009; Muse et al., 2007; Zeitlinger et al., 2007), it has been hypothesized that promoters contribute to the timing and synchronicity of transcription (Lagha et al., 2013) (Fig. 6). Using quantitative imaging of reporter constructs with different promoters and enhancers active during cellularization, Lagha et al. (2013) concluded that minimal promoter sequences are sufficient to direct synchronous expression from a given promoter between cells; this is thought to be coordinated by Pol II pausing and to be important for normal development. Changing the degree of synchrony in a computational model generates gastrulation defects, similar to those observed experimentally, suggesting that synchrony is key for developmental progression.

The above study also highlights the link between the timing and level of transcription. Promoters that differ in paused Pol II occupancy also lead to changes in the number of transcripts produced per cell. This can be explained by the effect on synchrony: promoters with more Pol II are activated earlier, which allows cells to produce and accumulate transcripts for a longer time, leading to higher levels. In *Drosophila* cell culture, Pol II pausing also inhibits new initiation between transcriptional bursts (Shao and Zeitlinger, 2017). This suggests that Pol II pausing prevents immediate reactivation after a transcriptional burst, which may increase transcriptional precision (i.e. reduce noise). It remains to be determined whether this also applies for Pol II-mediated regulation of transcription in the context of the developing embryo.

Transcription factories in which active Pol II is clustered in the nucleus may also affect transcript levels. A theoretical model of Pol II clustering in mammalian cells, fitted to data from super-resolution microscopy, showed that Pol II clusters act to increase transcript number not by increasing burst frequency or amplitude but by increasing burst duration (Cho et al., 2016). This would make the role of Pol II clusters complementary to the role of enhancers, which so far have been implicated in modulating burst frequency (Fukaya et al., 2016).

#### Enhancers coordinate temporal expression

Enhancers can also regulate the timing of gene expression during development, both at the level of a single enhancer operating on its cognate promoter, and at the level of multiple enhancers together regulating a single promoter (Long et al., 2016). For example, the *brk* locus is regulated by two enhancers in the *Drosophila* early embryo; the downstream enhancer activates expression early in the early embryo, whereas the upstream enhancer drives expression later, during cellularization and gastrulation (Dunipace et al., 2013). It has been proposed that an autoregulatory mechanism allows *brk* to switch from being activated by one enhancer to the other. A promoter-proximal element required for activation by either enhancer contains Brk protein binding sites. As the level of Brk increases, it binds the promoter-proximal element and mediates the switch to activation by the upstream enhancer and a subsequent change in the expression pattern. This mechanism shows that at least some shadow enhancers do not activate simultaneously, and can in fact act as a mechanism to coordinate expression in time.

#### Pioneer factors coordinate temporal expression

Changes in chromatin state, especially at regulatory elements such as promoters and enhancers, play a significant role in regulating both the timing and specificity of transcription during embryogenesis (Cantone and Fisher, 2013; Perino and Veenstra, 2016). One of the most striking examples of global synchronized transcriptional activation occurs during the maternal to zygotic transition (MZT) (Lee et al., 2014). After fertilization, embryos are transcriptionally silent and development is mostly controlled by maternally contributed factors. During the MZT, maternal factors are degraded while the zygotic transcription of thousands of genes ensues, taking control over development. This process is associated with stepwise changes in the chromatin landscape both at enhancers and promoters (Boija and Mannervik, 2015; Cantone and Fisher, 2013; Hontelez et al., 2015; Hug et al., 2017; Li et al., 2014). In *Drosophila*, the TF Zelda (also known as Vielfaltig) is known to reshape the chromatin landscape at this critical developmental stage, regulating the transcriptional activation and temporal coordination of a substantial subset of early embryonic genes (Foo et al., 2014; Harrison et al., 2011; Liang et al., 2008; Nien et al., 2011; Schulz et al., 2015; Sun et al., 2015). Zelda has thus been proposed to function as a pioneer factor – a specialized type of TF that is known to bind nucleosomal DNA and form open chromatin (Iwafuchi-Doi and Zaret, 2016; Zaret and Mango, 2016). However, the mechanism by which Zelda and other pioneer factors form open chromatin and regulate transcriptional activation is incompletely understood (Swinstead et al., 2016). Several studies have confirmed that Zelda acts as a transcriptional ʻswitch' to activate the expression of zygotic genes (Crocker et al., 2017a; Sun et al., 2015). These studies have converged on a qualitative model for the role of Zelda, wherein the protein acts exclusively by remodeling chromatin from a ʻclosed' to ʻopen' state to allow TF binding and gene activation, but this has not yet been translated into a quantitative model built from the underlying assumptions.

## Perspectives

In this Review, we sought to define a useful vocabulary for discussing quantitative features of transcription, namely the precision and accuracy of the location, levels and timing of transcription. We discussed recent selected work in *Drosophila* embryos that has attempted to decipher the mechanisms that impinge upon these features. Our summary is in no way comprehensive; indeed, work in other model systems has provided much to the field and our own thinking. We restricted our discussion to the *Drosophila* embryo, which has long served as a model for quantitative studies of transcription, because genetics, biochemistry and microscopy are well established for this model system and because many of the molecular players are known (Gregor et al., 2014).

Technological advances have provided new tools to further dissect the mechanisms that contribute to precise and accurate transcription. Measuring the timing, location and level of gene expression across a population of cells requires the use of quantitative tools, which are growing in use and scope within developmental biology. This provides an exceptional opportunity for the use of mathematical models to guide the interpretation of quantitative data and the design of further experiments. It will also be worthwhile to contrast mechanistically motivated models, like those we have discussed here, with statistical models, which are widely employed to interpret functional genomics data (Kim et al., 2009). Statistical models are required to draw any meaningful inferences from large data sets and can provide insights into overall trends and correlations within such data. The models we argue for here take a complementary approach and use dynamical data to seek the underlying molecular interactions of transcriptional regulation in well-characterized model systems. Compared with the vast array of tools and statistical frameworks built for functional genomics data, the mechanistically motivated theoretical models that lend predictive understanding from quantitative data are less developed. Although the theoretical frameworks that have proved to be successful in describing prokaryotic transcription may be built on assumptions that do not apply in higher organisms, their success signals that they provide a good starting point in engineering new frameworks. Gaining a deeper and more comprehensive understanding of transcriptional regulation will thus depend on our efforts to couple increasingly complex quantitative data with insightful modeling frameworks.
